# Analysis of the effect of clustered nursing interventions on enteral nutrition in critically ill stroke patients

**DOI:** 10.1097/MD.0000000000045381

**Published:** 2025-10-31

**Authors:** Yidi Chen, Yulan Luo, Yongming Tian

**Affiliations:** a Department of Critical Care Medicine, West China Hospital, Sichuan University/West China School of Nursing, Sichuan University, Chengdu, Sichuan Province, China.

**Keywords:** clustered nursing, critically ill patients, enteral nutrition, gastrointestinal tolerance, nutritional risk score, nutritional support, stroke

## Abstract

This study aims to investigate the impact of clustered nursing interventions on the implementation of enteral nutrition (EN) in critically ill stroke patients and provide evidence for optimizing nutritional management in this patient population. This single-center retrospective cohort study included 83 critically ill stroke patients admitted to the intensive care unit (ICU) of our hospital from February 2023 to June 2024. Patients were divided into a routine care group (n = 47) and a clustered care group (n = 36) based on different nursing intervention strategies. To minimize confounding bias, propensity score matching was performed, and 36 patients were included in each group for final analysis. Nutritional indicators (albumin, total protein, prealbumin, cholinesterase), Nutritional Risk Screening 2002 (NRS2002), achievement of EN targets, gastrointestinal (GI) adverse events, GI tolerance, and short-term clinical outcomes (ICU stay and total hospital stay) were compared between the 2 groups. Baseline characteristics were balanced between the 2 groups after matching. Compared with the routine care group, the clustered care group showed significantly higher nutritional indicators (albumin, total protein, prealbumin, cholinesterase) on days 3 and 7 of intervention (*P* < .001); NRS2002 scores were significantly lower on days 7 and 14 (*P* < .001). The clustered care group achieved EN targets in a shorter time, with higher rates of energy and protein target attainment (*P* < .01). The incidence of GI adverse events such as gastric retention, abdominal distension, vomiting, and constipation was significantly reduced (*P* < .05), and GI tolerance was significantly better (83.3% vs 61.1%, *P* = .028). In terms of short-term outcomes, the clustered care group had significantly shorter ICU and total hospital stays (*P* < .01). Clustered nursing interventions can effectively improve nutritional status, enhance EN target attainment, improve GI tolerance, and shorten hospital stay in critically ill stroke patients. These findings support the clinical value of promoting such interventions in critical care nursing practice.

## 1. Introduction

Stroke is one of the leading causes of disability and death worldwide, particularly in China, where both its mortality and disability rates remain alarmingly high, posing a serious threat to public health and quality of life.^[1]^ Critically ill stroke patients, who often suffer from extensive neurological damage and multi-organ dysfunction, present considerable clinical challenges and account for approximately 80% of all stroke-related deaths.^[2]^ Among various aspects of critical care, nutritional support – especially enteral nutrition (EN) – is a key intervention influencing patient prognosis and recovery. EN not only helps meet patients’ metabolic demands and maintain gastrointestinal (GI) barrier function, but also reduces the risk of infections, shortens hospital stays, and lowers medical costs.^[3]^

Although current guidelines recommend initiating EN within 48 hours of hospital admission for critically ill patients,^[4]^ in clinical practice, stroke patients in critical condition frequently experience complications such as impaired consciousness, dysphagia, and GI dysfunction. These factors significantly increase the risk of nutritional intolerance, gastric retention, abdominal distension, constipation, and aspiration, resulting in low implementation and target achievement rates of EN, which may negatively affect treatment outcomes and overall prognosis.^[5,6]^ Existing studies have also shown that EN management under routine nursing care is often passively executed based on physician orders, lacking systematic, individualized assessment and dynamic optimization. As a result, the quality of nutritional support remains inconsistent and often suboptimal.^[7,8]^

In recent years, clustered care intervention has emerged as a promising nursing model in critical care, characterized by the coordinated application of multiple evidence-based measures and standardized procedures.^[9,10]^ This approach emphasizes multidisciplinary collaboration and evidence-guided protocols, aiming to enhance both the consistency and individualization of interventions. Previous literature has demonstrated the effectiveness of bundled care in preventing ventilator-associated pneumonia, deep vein thrombosis, and pressure ulcers.^[11,12]^ However, research on its application in the EN management of critically ill stroke patients remains limited.

Based on prior nursing practice and evidence-based principles, this study developed a bundled nursing intervention strategy centered on the entire process of EN. The strategy includes key components such as team training, nutritional assessment, protocol optimization, complication prevention, and enhancement of GI tolerance. The aim is to evaluate the impact of this intervention on EN target achievement, nutritional status, GI tolerance, and short-term clinical outcomes in critically ill stroke patients. This study seeks to explore a scientific and efficient nutritional care pathway for critically ill patients, and to provide evidence-based support for the implementation of systematic and refined EN care in clinical practice.

## 2. Materials and methods

### 2.1. Study design

This study was conducted in accordance with the principles of the Declaration of Helsinki. Ethical approval was obtained from the Ethics Committee of West China Hospital, Sichuan University (Approval No. 2023-ICU-EN-041, approved on January 15, 2023). This study was a single-center retrospective cohort study. Eligible patients were identified through the hospital’s electronic medical record system and nursing information system (NIS), and retrospective data analysis was conducted. A total of 83 critically ill stroke patients admitted to the intensive care unit (ICU) of our hospital between February 2023 and June 2024 were included. Based on the type of nursing intervention received, patients were divided into a routine care group (n = 47) and a bundled care group (n = 36). To reduce the impact of confounding factors on study outcomes, propensity score matching (PSM) was employed. Patients were matched 1:1 based on baseline characteristics including age, sex, type of stroke, severity scores (e.g., GCS, National Institutes of Health Stroke Scale [NIHSS]), and comorbidities. After matching, 36 patients remained in each group and were included in the final analysis.

### 2.2. Inclusion and exclusion criteria

Inclusion criteria: Age ≥ 18 years; met diagnostic criteria for stroke according to the Chinese Guidelines for the Prevention and Treatment of Stroke, with radiological confirmation; received EN support within 48 hours of admission, maintained for at least 7 days; met the criteria for critical illness (GCS ≤ 12, or NIHSS ≥ 10, or required mechanical ventilation); hospital stay ≥ 7 days; and complete records of nursing interventions and nutritional support.

Exclusion criteria: coexisting severe GI diseases (e.g., obstruction, bleeding, fistula); malignancies or end-stage organ failure; hospital stay <7 days or voluntary discharge; changes in treatment plan during nutrition support leading to incomplete intervention; other special conditions such as pregnancy or immunodeficiency that may affect nutritional metabolism; and Missing or incomplete clinical data.

## 3. Standard nursing procedures

### 3.1. *Routine care*^[13]^

#### 3.1.1. Management of enteral nutrition access

Strict aseptic techniques were followed during all procedures. After appropriate nasogastric tube placement, the tube was securely fixed using adhesive dressings or medical tape to prevent dislodgement or unplanned removal. Nursing staff conducted frequent rounds to inspect tube patency and fixation status, with at least 2 checks per shift. Daily nasal care was provided, including regular lubrication to minimize friction between the tube and nasal mucosa, thereby preventing bleeding and pressure injuries.

#### 3.1.2. Enteral nutrition administration

EN was administered based on physician orders, with controlled infusion rates starting at 25 to 50 mL/h and gradually increased to avoid adverse reactions from rapid infusion. Nutritional solutions were maintained at a constant temperature (approximately 37°C) and delivered via gravity infusion or infusion pumps. Gastric residual volume (GRV) was monitored every 4 to 6 hours; if GRV exceeded 200 mL, feeding was paused and reported to the attending physician.

#### 3.1.3. Positioning and basic care

During feeding, patients were assisted into a lateral position or had the head of the bed elevated to 30° to 45°, maintained for at least 30 minutes. Patients were repositioned every hour to relieve pressure, with special attention to protecting bony prominences using soft pads. Skin was kept clean and dry to prevent pressure ulcers.

#### 3.1.4. Monitoring and management of complications

Patients were closely monitored for signs of GI intolerance, including abdominal distension, diarrhea, constipation, and aspiration. Any abnormalities were addressed promptly. Prokinetic agents and proton pump inhibitors were used as prescribed. Drainage tubes were routinely cared for to ensure patency and prevent infection or blockage.

#### 3.1.5. Infection prevention and staff training

Strict aseptic techniques were maintained during all procedures to prevent nosocomial infections. Regular training sessions on EN were conducted to enhance nursing staff’s adherence to standardized procedures and raise awareness of risk prevention.

### 3.2. *Bundled nursing interventions*^[14]^

Patients in the observation group received systematic and standardized bundled nursing interventions in addition to routine care. The specific measures included the following.

#### 3.2.1. Nursing team training and assessment enhancement

A specialized training program on EN was conducted for the critical care nursing team. The training covered the importance of early EN, implementation procedures, and prevention of related complications. Nursing staff were required to become proficient in bundled nursing protocols, infusion techniques, and GI function assessment. The training aimed to enhance the overall competence and awareness of the nursing team regarding EN support, ensuring consistency and standardization in nursing interventions.

#### 3.2.2. Development and optimization of individualized nutrition plans

Clinicians and dietitians collaboratively formulated individualized EN plans based on the patient’s condition, nutritional risk screening results, and metabolic needs. Appropriate formulas were selected, prioritizing low-osmolality, peptide-based, or immune-enhancing formulas to meet energy and protein requirements. Nutritional solutions were prepared and managed by designated personnel using aseptic techniques, with fresh preparation before each use to avoid contamination. Solution temperature was maintained between 37 and 42°C to prevent GI irritation and intolerance caused by excessive heat or cold.

#### 3.2.3. Optimization of nutrition infusion protocols

EN infusion followed a principle of “low to high concentration, slow to fast rate,” delivered continuously via an EN pump. The initial infusion rate was set at 40 to 50 mL/h and gradually increased to 80 to 100 mL/h depending on GI tolerance. GRV was assessed every 4 hours, and the infusion plan was adjusted if GRV exceeded 100 mL. The osmolality of the nutritional solution was strictly controlled within 279 to 330 mmol/L to prevent osmotic diarrhea.

#### 3.2.4. Complication prevention and intervention

GI function was closely monitored, and GI prokinetic drugs were avoided unless necessary. For patients with abdominal distension or constipation, individualized adjustments were made to the EN formula. Dietary fiber supplementation was standardized, with soluble fiber 10 to 15 g/d added to the enteral formula in cases of reduced intestinal motility or constipation, unless contraindicated. Probiotics were administered according to the hospital’s nutrition support protocol, with a daily dose equivalent to 1 × 10^9^ colony-forming unit for at least 7 consecutive days in patients presenting with diarrhea or suspected intestinal dysbiosis. All supplementation was supervised by the attending physician and recorded in the NIS to ensure consistency. Aseptic techniques were strictly followed during nutritional support to reduce the risk of iatrogenic infection and diarrhea.

#### 3.2.5. Respiratory management

For patients requiring mechanical ventilation or airway management, airway humidification was maintained, and sputum was suctioned regularly. Nebulization or mechanical sputum clearance was used when necessary to prevent secretion retention and respiratory infection. High-flow oxygen was administered for 2 minutes before suctioning, and gentle technique was employed to avoid hypoxia. Oral secretions were cleared daily to maintain airway hygiene and reduce the risk of aspiration pneumonia.

#### 3.2.6. Skin care and pressure ulcer prevention

Skin care was reinforced, particularly in the perianal area, which was kept clean and dry. After defecation, the area was promptly cleaned, and protective agents or talcum powder were applied when needed to prevent irritation, redness, or erosion. Bedding was kept clean, dry, and wrinkle-free. Patients were repositioned every 2 hours to relieve pressure on local areas and prevent pressure ulcers.

#### 3.2.7. Comprehensive assessment and dynamic adjustment

The nursing team performed daily assessments of patients’ nutritional status, GI tolerance, and complication occurrences. Nutrition support plans were dynamically adjusted based on assessment results. The effects of nursing interventions and patient responses were documented in a timely manner to ensure continuity and effectiveness of nutritional therapy, promote target achievement in EN, and improve clinical outcomes.

## 4. Data collection

All data collection procedures strictly adhered to de-identification protocols to ensure patient privacy. Personally identifiable information (e.g., name, hospital ID, national ID number) was removed, and each enrolled patient was assigned a unique code for study purposes. Data collection was conducted jointly by 2 research nurses who had received standardized training. A double-check system was implemented for both data collection and entry to ensure accuracy. Final datasets were independently reviewed and locked by data analysts. The entire process of data collection and management complied with the Declaration of Helsinki and the relevant guidelines of the hospital’s ethics committee.

### 4.1. General information and baseline characteristics

Demographic and clinical baseline characteristics were collected for all enrolled patients, including age, sex, body mass index, stroke type (ischemic or hemorrhagic), comorbidities (e.g., hypertension, diabetes, coronary artery disease), and severity scores (NIHSS,^[15]^ GCS [Glasgow Coma Scale],^[16]^ APACHE II [Acute Physiology and Chronic Health Evaluation II),^[17]^ and SOFA [sequential organ failure assessment]^[18]^). Stroke types were confirmed by cranial computed tomography or magnetic resonance imaging. NIHSS and GCS scores were recorded during the initial disease course, while APACHE II and SOFA scores were assessed within 24 hours of ICU admission by the attending physician. Comorbidities were diagnosed based on medical history review and laboratory test results.

### 4.2. Measurement of nutrition-related indicators

Nutritional indicators were dynamically monitored, including serum albumin (g/L), total protein (g/L), prealbumin (mg/L), and cholinesterase (U/L). Blood samples (5 mL of venous blood) were collected on days 1, 3, and 7 of the intervention. After centrifugation, all samples were processed in the hospital’s clinical laboratory using standardized procedures that complied with laboratory quality control protocols.

### 4.3. Nutritional risk assessment

Nutritional risk was assessed using the Nutritional Risk Screening 2002 (NRS2002) tool.^[19]^ The score includes recent weight loss, reduced dietary intake, disease severity, and an age-related adjustment (+1 point for patients ≥70 years old). A total score ≥3 indicates the presence of nutritional risk. Assessments were conducted at 3 time points: before the intervention, on day 7, and on day 14 of the intervention. Two trained nursing staff independently performed the assessments, and the average of their scores was recorded.

### 4.4. Documentation of enteral nutrition target achievement

EN targets were based on patients’ actual or adjusted body weight, with goals set at ≥25 kcal/kg/d for energy intake and ≥1.2 g/kg/d for protein intake. The number of days required to reach target energy and protein intake was recorded. The target achievement rate within 7 days of nutritional support was also calculated. EN infusion data were extracted from the NIS, with daily verification of total intake to ensure data completeness.

### 4.5. Monitoring of gastrointestinal tolerance and adverse reactions

GI adverse events monitored included gastric retention (≥200 mL), abdominal distension, diarrhea (≥3 times/d), vomiting, and constipation. GRV was measured via nasogastric tube aspiration every 4 hours. Criteria for diarrhea and constipation were based on clinical nursing standards and recorded by bedside nurses. Good GI tolerance was defined as the absence of any GI adverse events. All data were recorded daily and reviewed by designated personnel.

### 4.6. Short-term prognostic indicators

Short-term outcome indicators included ICU length of stay (days) and total hospital length of stay (days). ICU stay was defined as the duration from ICU admission to discharge from the ICU. Total hospital stay referred to the number of days from initial admission to discharge. Relevant data were obtained from the medical records department and verified by the nursing team before inclusion in the analysis.

## 5. Statistical analysis

Data analysis was performed using SPSS version 26.0 (IBM Corporation, Armonk). Continuous variables were expressed as mean ± standard deviation (*x̄* ± *s*), and comparisons between groups were conducted using the independent samples *t*-test. Categorical variables were presented as frequencies and percentages, and between-group comparisons were performed using the χ² test or Fisher’s exact test, as appropriate. PSM was used to minimize confounding, with 1:1 nearest-neighbor matching based on variables such as age, sex, stroke type, NIHSS score, and GCS score. The caliper was set at 0.02. Repeated measures data were analyzed using repeated measures analysis of variance (ANOVA) to evaluate time effects, group effects, and interaction effects. A 2-sided *P*-value < .05 was considered statistically significant, with α = 0.05 as the threshold for significance. The proportion of missing data in this study was <5% across all variables. Multiple imputation was applied to handle missing values, ensuring data completeness and the robustness of the statistical analysis.

## 6. Results

### 6.1. Comparison of baseline characteristics between the 2 groups

A total of 83 critically ill stroke patients were included in the study, with 47 patients in the routine care group and 36 in the bundled care group. Before PSM, there were significant differences between the 2 groups in terms of age, stroke type, NIHSS score, GCS score, presence of hypertension, diabetes, serum albumin level, time to initiation of EN, APACHE II score, and SOFA score (*P* < .05).

After 1:1 PSM, 36 patients were included in each group. Post-matching, the mean age was 66.9 ± 9.8 years in the routine care group and 65.7 ± 9.4 years in the bundled care group (*P* = .512). The NIHSS scores were 15.4 ± 3.3 and 14.5 ± 3.5, respectively (*P* = .243); serum albumin levels were 31.0 ± 4.5 g/L and 31.7 ± 4.6 g/L (*P* = .429); and time to initiation of EN was 26.5 ± 7.1 hours and 24.3 ± 6.2 hours (*P* = .228). No statistically significant differences were observed in other baseline characteristics (*P* > .05), indicating that the 2 groups were balanced and comparable (see Table 1).

**Table 1:** Baseline characteristics of patients before and after propensity score matching (PSM).

Variables	Before matching		*P*-value	After matching		*P*-value
	Routine care group (n = 47)	Bundle care group (n = 36)		Routine care group (n = 36)	Bundle care group (n = 36)	
Age (yr)	71.3 ± 8.9	65.7 ± 9.4	**.003**	66.9 ± 9.8	65.7 ± 9.4	.512
Sex (male/female)	33/ 14	21/ 15	.164	22/ 14	21/ 15	.815
Stroke type (ischemic/hemorrhagic)	32/ 15	19/ 17	**.047**	21/ 15	19/ 17	.678
NIHSS score (points)	17.1 ± 3.2	14.5 ± 3.5	**<.001**	15.4 ± 3.3	14.5 ± 3.5	.243
GCS score (points)	7.8 ± 1.4	8.6 ± 1.7	**.01**	8.4 ± 1.5	8.6 ± 1.7	.622
Hypertension (n, %)	39 (83.0%)	22 (61.1%)	**.028**	27 (75.0%)	22 (61.1%)	.199
Diabetes mellitus (n, %)	18 (38.3%)	7 (19.4%)	**.049**	9 (25.0%)	7 (19.4%)	.564
Coronary heart disease (n, %)	15 (31.9%)	6 (16.7%)	.108	7 (19.4%)	6 (16.7%)	.755
BMI (kg/m²)	22.9 ± 3.2	24.1 ± 3.5	.082	23.7 ± 3.1	24.1 ± 3.5	.558
Serum albumin (g/L)	29.1 ± 4.2	31.7 ± 4.6	**.004**	31.0 ± 4.5	31.7 ± 4.6	.429
Time to enteral nutrition initiation (h)	42.6 ± 5.4	24.3 ± 6.2	**<.001**	26.5 ± 7.1	24.3 ± 6.2	.228
APACHE II score	20.7 ± 5.3	17.4 ± 4.8	**.002**	18.2 ± 4.9	17.4 ± 4.8	.478
SOFA score	7.8 ± 2.3	6.3 ± 2.1	**.006**	6.8 ± 2.2	6.3 ± 2.1	.384
Infection [n (%)]	28 (59.6%)	13 (36.1%)	**.024**	15 (41.7%)	13 (36.1%)	.628
Hepatic/renal dysfunction (n, %)	19 (40.4%)	9 (25.0%)	.13	11 (30.6%)	9 (25.0%)	.593
Gastrointestinal dysfunction (n, %)	26 (55.3%)	12 (33.3%)	**.041**	15 (41.7%)	12 (33.3%)	.469

Bold values indicate statistical significance.

APACHE II = Acute Physiology and Chronic Health Evaluation II, GCS = Glasgow Coma Scale, NIHSS = National Institutes of Health Stroke Scale, PSM = propensity score matching, SOFA = sequential organ failure assessment.

### 6.2. Effect of bundled nursing on nutritional indicators

To evaluate the impact of bundled nursing interventions on the nutritional status of critically ill stroke patients, dynamic monitoring and analysis were conducted on serum levels of albumin, total protein, prealbumin, and cholinesterase in both groups. The analysis revealed significant differences in the time effect, group effect, and time-by-group interaction effect for all 4 nutritional indicators (*P* < .01), as shown in Table 2.

**Table 2:** Changes in nutritional indicators over time between 2 groups.

Indicator	Group	Day 1 (Mean ± SD)	Day 3 (Mean ± SD)	Day 7 (Mean ± SD)	Time effect *F*/*P*	Group effect *F*/*P*	Time × Group effect *F*/*P*
Albumin (g/L)	Routine Care	30.25 ± 4.60	29.10 ± 4.75	28.05 ± 4.30	16.524/<.01	14.310/<.01	13.270/<.01
	Bundle Care	30.10 ± 4.55	33.85 ± 4.20	37.20 ± 4.00			
	*t*	0.126	4.225	7.034			
	*P*	.9	<.001	<.001			
Total protein (g/L)	Routine Care	58.80 ± 6.10	57.20 ± 6.00	55.90 ± 5.80	15.423/<.01	12.875/<.01	11.540/<.01
	Bundle Care	58.65 ± 6.20	62.75 ± 5.90	65.40 ± 5.50			
	*t*	0.11	4.06	7.118			
	*P*	.913	<0.001	<.001			
Prealbumin (mg/L)	Routine Care	185.50 ± 42.30	178.20 ± 40.60	170.80 ± 39.10	19.005/<.01	17.460/<.01	15.890/<.01
	Bundle Care	186.00 ± 43.10	205.80 ± 41.50	230.40 ± 40.20			
	*t*	0.053	3.115	6.012			
	*P*	.958	.003	<.001			
Cholinesterase (U/L)	Routine Care	4100 ± 650	3950 ± 620	3800 ± 600	20.850/<.01	18.275/<.01	16.690/<.01
	Bundle Care	4120 ± 660	4400 ± 630	4750 ± 610			
	*t*	0.135	3.421	5.84			
	*P*	.893	.001	<.001			

SD = standard deviation.

At baseline (day 1 of intervention), there were no significant differences between the 2 groups in any of the nutritional indicators (*P* > .05), indicating good comparability. However, as the intervention progressed, patients in the bundled care group demonstrated significantly better improvements. By day 3, the albumin level in the bundled care group was markedly higher (33.85 ± 4.20 g/L) compared to the routine care group (29.10 ± 4.75 g/L, *P* < .001). Similar improvement trends were observed for total protein, prealbumin, and cholinesterase levels (*P* < .01).

By day 7, the nutritional benefits of the bundled care intervention became even more pronounced. The albumin level in the bundled care group reached 37.20 ± 4.00 g/L, significantly higher than that in the control group (28.05 ± 4.30 g/L). Likewise, total protein (65.40 ± 5.50 g/L vs 55.90 ± 5.80 g/L), prealbumin, and cholinesterase levels were all significantly improved in the bundled care group compared to the control group (*P* < .001).

### 6.3. Comparison of NRS2002 scores between the two groups

To evaluate the effect of bundled nursing interventions on nutritional risk in critically ill stroke patients, NRS2002 scores were compared between the 2 groups at baseline, day 7, and day 14 of the intervention (see Table 3).

**Table 3:** Comparison of NRS2002 scores between the 2 groups at different time points.

Group	Before intervention	7 d after intervention	14 d after intervention	Time effect *F*/*P*	Group effect *F*/*P*	Interaction effect *F*/*P*
Control group (n = 36)	4.75 ± 0.85	3.90 ± 0.80	3.30 ± 0.70	48.725/<001	22.543/<.001	19.320/<.001
Bundle care group (n = 36)	4.80 ± 0.82	3.15 ± 0.75	2.40 ± 0.65			
*t* value	0.258	4.123	6.214			
*P*-value	.797	<.001	<.001			

NRS2002 = Nutritional Risk Screening 2002.

At baseline, there was no statistically significant difference in NRS2002 scores between the 2 groups (control group: 4.75 ± 0.85; bundled care group: 4.80 ± 0.82; *P* = .797), indicating comparability. However, after 7 days of intervention, the NRS2002 score in the bundled care group was significantly lower than that in the control group (3.15 ± 0.75 vs 3.90 ± 0.80; *P* < .001). By day 14, the score in the bundled care group had further decreased to 2.40 ± 0.65, which was also significantly lower than that in the control group (3.30 ± 0.70; *P* < .001).

Repeated measures ANOVA revealed statistically significant differences in time effect (*F* = 48.725, *P* < .001), group effect (*F* = 22.543, *P* < .001), and the interaction between time and group (*F* = 19.320, *P* < .001), indicating that bundled nursing interventions effectively reduced nutritional risk over time.

### 6.4. Comparison of enteral nutrition target achievement between the 2 groups

To assess the impact of bundled nursing interventions on EN target achievement in critically ill stroke patients, this study compared the time to reach target energy and protein intake, as well as the respective achievement rates between the 2 groups (see Table 4).

**Table 4:** Comparison of enteral nutrition target achievement between 2 groups.

Indicator	Control group (n = 36)	Bundle care group (n = 36)	*t*/χ² Value	*P*-value
Time to achieve target energy (d)	6.2 ± 1.5	4.1 ± 1.3	6.472	<.001
Time to achieve target protein intake (d)	7.0 ± 1.7	5.0 ± 1.4	5.982	<.001
Energy support achievement rate (≥25 kcal/kg/d)	58.3% (21/36)	83.3% (30/36)	6.667	.01
Protein support achievement rate (≥1.2 g/kg/d)	52.8% (19/36)	80.6% (29/36)	7.68	.006

The results showed that the time required to reach the target energy intake (≥25 kcal/kg/d) was significantly shorter in the bundled care group than in the control group (4.1 ± 1.3 days vs 6.2 ± 1.5 days, *P* < .001). Similarly, the time to achieve the target protein intake (≥1.2 g/kg/d) was significantly reduced in the bundled care group (5.0 ± 1.4 days vs 7.0 ± 1.7 days, *P* < .001).

In addition, the bundled care group demonstrated significantly higher rates of achieving energy support targets (83.3% vs 58.3%, *P* = .01) and protein support targets (80.6% vs 52.8%, *P* = .006) compared to the control group.

### 6.5. Comparison of gastrointestinal tolerance and adverse reactions between the 2 groups

To evaluate the impact of bundled nursing interventions on GI tolerance during EN in critically ill stroke patients, this study compared the incidence of GI adverse reactions and overall GI tolerance rates between the 2 groups (see Table 5).

**Table 5:** Comparison of gastrointestinal tolerance and adverse reactions between 2 groups.

Gastrointestinal adverse reactions	Control group (n = 36)	Bundle care group (n = 36)	χ²/*t* value	*P*-value
Gastric retention (≥ 200 mL) (n, %)	12 (33.3%)	5 (13.9%)	4.05	.044
Abdominal distension (n, %)	14 (38.9%)	6 (16.7%)	4.8	.028
Diarrhea (n, %)	10 (27.8%)	4 (11.1%)	3.333	.068
Vomiting (n, %)	8 (22.2%)	2 (5.6%)	4	.046
Constipation (n, %)	15 (41.7%)	7 (19.4%)	4.773	.029
Gastrointestinal tolerance rate (%)	61.1% (22/36)	83.3% (30/36)	4.8	.028

Compared with the control group, the bundled care group showed a significantly lower incidence of gastric retention (13.9% vs 33.3%, *P* = .044) and abdominal distension (16.7% vs 38.9%, *P* = .028). In addition, the incidence of vomiting was significantly reduced in the bundled care group (5.6% vs 22.2%, *P* = .046). Although the occurrence of diarrhea was lower in the bundled care group (11.1% vs 27.8%), the difference did not reach statistical significance (*P* = .068). The incidence of constipation was significantly lower in the bundled care group (19.4% vs 41.7%, *P* = .029). Overall, the GI tolerance rate was significantly higher in the bundled care group compared to the control group (83.3% vs 61.1%, *P* = .028).

### 6.6. Comparison of short-term clinical outcomes between the 2 groups

To further evaluate the impact of bundled nursing interventions on the short-term clinical outcomes of critically ill stroke patients, this study compared ICU length of stay and total hospital stay between the 2 groups (see Table 6).

**Table 6:** Comparison of short-term prognostic indicators between the 2 groups.

Indicators	Control group (n = 36)	Bundle care group (n = 36)	*t* value	*P*-value
ICU length of stay (d)	12.5 ± 3.6	9.8 ± 2.9	3.501	.001
Total hospital stay (d)	24.3 ± 5.5	18.7 ± 4.8	4.59	<.001

ICU = intensive care unit.

The results showed that the ICU length of stay was significantly shorter in the bundled care group compared to the control group (9.8 ± 2.9 days vs 12.5 ± 3.6 days, *P* = .001). Likewise, the total hospital stay was also significantly reduced in the bundled care group (18.7 ± 4.8 days vs 24.3 ± 5.5 days, *P* < .001).

## 7. Discussion

Critically ill stroke patients often suffer from malnutrition due to dysphagia, impaired consciousness, and a hypermetabolic state, which may further aggravate neurological damage and delay recovery. EN is a key strategy to improve the nutritional status of such patients; however, in clinical practice, low target achievement rates are frequently observed due to GI intolerance and non-standardized nursing practices, ultimately affecting patient outcomes. Bundled nursing interventions, which integrate multidisciplinary collaboration, standardized procedures, and dynamic assessment, may offer a systematic solution to these issues.^[20]^ In this retrospective cohort study, bundled nursing was found to significantly outperform routine care in improving nutritional indicators, enhancing EN tolerance, and shortening hospital stay. These effects may be attributed to optimized infusion management, enhanced complication prevention, and individualized intervention strategies.

## 12. Mechanisms and significance of nutritional improvement

This study demonstrated that nutritional indicators such as serum albumin and prealbumin improved significantly in the bundled care group as early as day 3 of intervention, with differences becoming more pronounced over time. This rapid improvement is likely associated with key elements of the bundled care protocol, including progressive infusion rate adjustments and individualized nutritional formula selection. Previous studies have indicated that stress responses in stroke patients can increase intestinal permeability, making them more susceptible to diarrhea and malabsorption when exposed to hyperosmolar or rapidly infused nutritional solutions.^[21]^ In this study, controlling the initial infusion rate (40–50 mL/h) and osmolarity (279–330 mmol/L), combined with the use of peptide-based or immune-enhancing formulas, may have minimized mucosal injury and improved protein absorption efficiency.

Furthermore, the bundled care protocol required GRV monitoring every 4 hours, with a threshold of 100 mL – lower than the 200 mL used in the routine care group – allowing for earlier detection of delayed gastric emptying and timely adjustment of the feeding strategy to avoid interruptions. This finding aligns with the “low-threshold intervention” strategy proposed by McClave et al,^[22]^ which demonstrated that strict GRV monitoring can improve EN target achievement rates by up to 20%.

It is noteworthy that the prealbumin level in the bundled care group increased by 23.9% from baseline by day 7, whereas it decreased by 7.9% in the routine care group. Prealbumin has a short half-life (2–3 days) and is considered a sensitive marker for evaluating the early effectiveness of nutritional interventions.^[23]^ This finding suggests that bundled nursing may exert its beneficial effects through 2 primary mechanisms: first, by maintaining constant temperature infusion (37–42°C), which helps preserve intestinal blood flow and reduces the risk of paralytic ileus caused by cold stimulation; and second, by adding probiotics to regulate the intestinal microbiota and improve the gut microenvironment. This is consistent with findings reported by Reignier et al,^[24]^ who observed that the combination of probiotics with EN in mechanically ventilated patients resulted in a 15% to 20% increase in prealbumin levels.

## 13. Optimization of enteral nutrition target achievement and gastrointestinal tolerance

In the bundled care group, the time required to achieve target energy and protein intake was shortened by 2 to 3 days compared to the routine care group, with target achievement rates improved by 25% to 30%. This advantage may be attributed to the use of continuous EN pump infusion combined with a “dynamic assessment-feedback” mechanism. Traditional gravity feeding often results in variable flow rates, which may lead to abdominal distension or gastric retention. In contrast, enteral feeding pumps allow precise control of infusion speed, thereby reducing GI burden.

This study also found that the incidence of gastric retention (13.9% vs 33.3%) and abdominal distension (16.7% vs 38.9%) was significantly lower in the bundled care group, consistent with findings from a multicenter study by Heyland et al^[25]^ Possible mechanisms include: maintaining the head of the bed at 30° to 45° to reduce the risk of reflux; and prioritizing the use of dietary fiber over laxatives in patients with constipation to avoid intestinal dysregulation (constipation occurred in only 19.4% of patients in the bundled care group vs 41.7% in the routine group).

Although the incidence of diarrhea in the bundled care group did not reach statistical significance (11.1% vs 27.8%, *P* = .068), the absolute risk reduction of 16.7% may be clinically meaningful and potentially limited by sample size. This trend aligns with findings from Khalid et al,^[26]^ who identified contamination of nutritional solutions as a major cause of diarrhea in critically ill patients. In the present study, the use of “freshly prepared” formulas and designated personnel for preparation and management may have reduced the risk of contamination.

## 14. Potential mechanisms for improved short-term prognosis

In the bundled care group, ICU length of stay and total hospital days were reduced by 2.7 and 5.6 days, respectively, which holds significant clinical and economic value. Beyond the direct benefits of improved nutritional status, components of the bundled care intervention – such as respiratory management and skin care – may have contributed to recovery by reducing complications.

For instance, the use of high-flow oxygen prior to suctioning and routine oral hygiene helped lower the incidence of aspiration pneumonia (13.9% in the bundled care group vs 27.8% in the routine care group, *P* = .12, pre-matching data), consistent with Kozeniecki et al,^[27]^ who reported that oral care can reduce the risk of ventilator-associated pneumonia. Additionally, turning patients every 2 hours and protecting the perianal skin reduced the incidence of pressure ulcers from 22.2% in the routine group to 5.6% in the bundled care group (*P* = .03), thereby decreasing the likelihood of secondary infections and prolonged hospitalization. Although clustered nursing interventions have been widely applied in critical care – for example, in the prevention of VPA, pressure ulcers, and deep vein thrombosis – their targeted use in the nutritional management of critically ill stroke patients has rarely been reported. The novelty of this study lies in extending the concept of clustered care to this high-risk population and systematically evaluating its effects on EN tolerance, nutritional status, and short-term clinical outcomes. By focusing on critically ill stroke patients, who often present with complex neurological impairment and GI dysfunction, our findings provide new evidence supporting the adaptation of clustered care strategies in a disease-specific context.

Furthermore, these results indicate that once standardized and protocolized, clustered nursing interventions could be feasibly incorporated into the routine workflow of ICU nursing. Embedding such evidence-based practices into daily care may enhance the consistency and quality of EN management, thereby improving patient outcomes in a reproducible manner.

## 15. Limitations and future directions

This study has several limitations. Although a retrospective cohort design supplemented with PSM was used to reduce baseline differences, residual confounding factors could not be completely eliminated. Uncontrolled variables such as the use of sedative drugs, mechanical ventilation parameters, or other unrecorded treatments may have interfered with GI function and influenced the results. Long-term neurological outcomes were not evaluated, and no follow-up data were available, which limits the ability to assess the sustained benefits of clustered care interventions. The relatively small sample size restricted subgroup analyses based on stroke subtypes (e.g., brainstem infarction), and no a priori power analysis was conducted, which may introduce the risk of positive bias. Although standardized criteria for probiotic and dietary fiber usage were applied, minor variations in individual patient responses and clinical discretion may still have affected implementation. As this was a single-center study with only 83 patients, the external validity of the findings is limited, and caution should be exercised when extrapolating to other regions or different ICU settings. Future studies should include multicenter randomized controlled trials with larger sample sizes, incorporate long-term follow-up data, and explore microbiome-based biomarkers to further clarify the gut–brain axis and its role in nutritional interventions for critically ill stroke patients.

## 16. Conclusion

Bundled nursing interventions – through standardized infusion protocols, early complication prevention, and multidisciplinary collaboration – were shown to significantly improve the nutritional status and short-term clinical outcomes of critically ill stroke patients. The main advantage of this model lies in translating evidence-based strategies into practical and operable nursing pathways while still maintaining individualized care. In addition to demonstrating clinical efficacy, this study also provides practical guidance for optimizing ICU nursing workflows. Incorporating clustered care into routine nursing protocols may serve as a valuable reference model to improve the standardization and efficiency of nutritional management in critically ill patients.

## Author contributions

**Conceptualization:** Yidi Chen, Yulan Luo, Yongming Tian.

**Data curation:** Yidi Chen, Yulan Luo, Yongming Tian.

**Formal analysis:** Yidi Chen, Yulan Luo, Yongming Tian.

**Investigation:** Yidi Chen, Yulan Luo, Yongming Tian.

**Methodology:** Yidi Chen, Yulan Luo, Yongming Tian.

**Supervision:** Yidi Chen, Yulan Luo, Yongming Tian.

**Validation:** Yidi Chen, Yulan Luo, Yongming Tian.

**Visualization:** Yidi Chen, Yulan Luo, Yongming Tian.

**Writing – original draft:** Yidi Chen, Yongming Tian.

**Writing – review & editing:** Yidi Chen, Yongming Tian.
